# Influencing factors and prognosis in patients with spontaneous intracerebral hemorrhage combined with pulmonary infection running head: pulmonary infection in ICH patients

**DOI:** 10.3389/fneur.2025.1644910

**Published:** 2025-11-06

**Authors:** Yan Liu, Chuyue Wu, Yu Huang, Jing Guo, Cuiping Du, Xin Li

**Affiliations:** 1 Department of Neurology, Chongqing University Three Gorges Hospital, Chongqing, China; 2 School of Medicine, Chongqing University, Chongqing, China; 3 Chongqing Municipality Clinical Research Center for Geriatric Diseases, Chongqing University Three Gorges Hospital, Chongqing, China

**Keywords:** spontaneous intracerebral hemorrhage, pulmonary infection, influencing factors, prognosis, stroke

## Abstract

**Background:**

Intracerebral hemorrhage (ICH) can lead to respiratory dysfunction and pulmonary infection (PI).

**Objectives:**

The present study aimed to investigate in-hospital factors influencing PIs in patients with spontaneous ICH and their prognosis.

**Methods:**

Clinical data of patients with spontaneous ICH were retrospectively collected from January 2021 to December 2022 to assess nosocomial consolidation of PIs, with follow-up evaluations for up to 1 year. The clinical factors influencing the development of PI were analyzed and their impact on prognosis was determined in patients with or without PI development.

**Results:**

A total of 864 patients with ICH were included in this study, of whom 568 (65.7%) had PIs. Independent factors influencing PIs included age, National Institute of Health Stroke Scale score at the time of admission, activities of daily living scale score at the time of admission, and C-reactive protein level (all *p* < 0.05). The adverse prognosis (70.8% vs. 39.5, 52.0% vs. 28.5, and 51.6% vs. 27.1%, respectively) and mortality rates (10.7% vs. 4.1, 6.5% vs. 1.8, and 10.3% vs. 3.2%, respectively) at the time of hospital discharge, 90 days after ICH onset, and 1 year after ICH onset were significantly higher in patients who developed PIs than in those who did not (*p* < 0.05).

**Conclusion:**

Pulmonary infection is a common complication of spontaneous ICH and may be influenced by patient age, length of hospital stay, and hospital admission status. Patients with spontaneous ICH and PI had worse prognoses and mortality rates than those without PI. Further clinical trial is necessary.

## Background

Intracerebral hemorrhage (ICH) is a type of stroke with a global prevalence of 37.6% and accounts for 10–20% of stroke patients (1–3). ICH is more disabling and lethal than ischemic stroke, causing approximately 50% of all stroke-related deaths (4, 5). ICH, as an acute cerebrovascular lesion, has a short onset and rapid progression, which can easily cause respiratory dysfunction (6) and aggravation of hypoxic brain tissues. As a result, ICH may result in secondary brain damage, and even poses a severe threat to the patient’s life and health (7).

Intracerebral hemorrhage is associated with a variety of complications, among which infection is very common and closely associated with poor prognosis (8). Among the patients with an ICH who have infection-related complications, 28% are pulmonary infections (PIs) (9, 10). PI has a great influence on the clinical outcome of patients with an ICH (9–11). PIs combined with an ICH are associated with a variety of risk factors, including advanced age, male gender, dysphagia, stroke-induced immunosuppression syndrome, a high National Institute of Health Stroke Scale (NIHSS) score, an elevated C-reactive protein (CRP) level, and chronic obstructive pulmonary disease (10, 12, 13). Most recent studies have focused on the correlation between systemic inflammatory response and stroke-associated pneumonia (SAP) (11, 14, 15). The incidences of SAP was very high, and the SAP mortality may rang between 10.1 and 37.3% among the mixed and stroke unit studies (11, 14, 15). PIs are the forms of SAP in ICH patients, and are often associated with an increase in morbidity, length of hospital stay, and mortality among patients with an ICH (9–11, 16).

However, the factors influencing PIs in patients with ICH in previous studies (8–16) varied and cannot be standardized. What are the differences in the prognosis and mortality between patients with ICH with and without PI? However, this clinical situation has not been reported frequently. Therefore, the factors influencing PIs complicating ICH as well as the influence of the presence or absence of PI on the prognosis of patients with ICH following discharge from the hospital were determined in this relatively large sample size study.

## Methods

### Study design

This retrospective cohort study was conducted at the Department of Neurology of our hospital. The research protocol was approved by the Clinical Trial Ethics Committee of the Chongqing University Three Gorges Hospital (No. KS-20230186). Written informed consent was obtained from all participants or their surrogates according to the Declaration of Helsinki (National Medical Research Registration and Archival Information System^1^ Unique identifiers: MR-50-23-001346 or MR-50-24-001923).

### Participants

**Figure 1:**
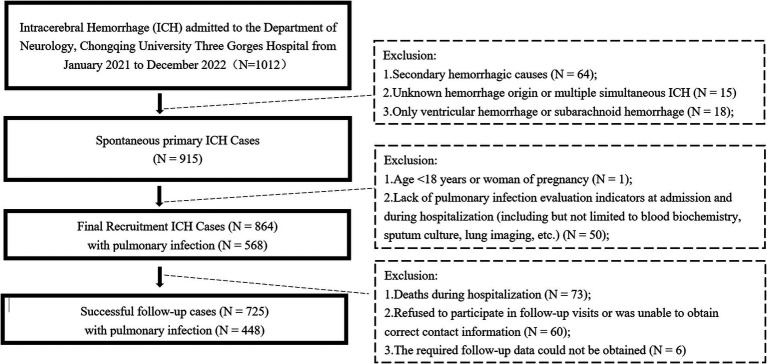
Flow diagram of recruitment of patients with intracerebral hemorrhage.

Patients with ICH were admitted to the Department of Neurology at the hospital between January 2021 and December 2022 were enrolled which included a period of COVID-19 outbreaks (from September 2022 to December 2022). The inclusion criteria were determined according to the 2023 Chinese Guidelines for the Diagnosis and Treatment of ICH, shown as follows: (1) >18 years of age and not pregnant; (2) the condition did not progress rapidly, and the patient died or was discharged >1 d after hospital admission; (3) PI evaluation indicators were recorded at the time of hospital admission and during the hospital stay, including but not limited to the blood biochemistry panel, sputum culture, and lung imaging; and (4) within 24 h from onset to admission. The exclusion criteria were as follows: (1) secondary hemorrhagic causes (e.g., tumor, trauma, cerebral infarction, and hemorrhagic transformation); (2) unknown origin of hemorrhage or multiple simultaneous ICHs; and (3) only ventricular or subarachnoid hemorrhage. The flow diagram of this study is shown in Figure 1.

### Clinical data

All the clinical data were collected from the medical record system, including demographic data (gender and age), treatment modality (including only medical and minimally invasive surgery [MIS] + medical), time in hours from the first CT to the onset of ICH (defined as the time from ICH onset or sudden aggravation of symptoms to the start of the first CT examination in the emergency department, as an outpatient, on as an inpatient), length of hospitalization in days, medical history (hypertension, diabetes, cigarette smoking, and alcohol consumption), admission data (height, weight, and vital signs), classification of Structural vascular lesions-Medications-Amyloid angiopathy-Systemic disease-Hypertension and Undetermined (SMASH-U), admission scores (including the modified Rankin scale [mRS] score before the ICH, the mRS score after the ICH, the Glasgow coma scale [GCS] score, and the NIHSS score), ICH-APS, NRS2002, water swallowing test, etc.

“Prognosis at 90 days” was defined as the primary outcome indicator in this study, and the rest indicators were recorded as secondary outcomes.

### Serum biochemical profile

The serum biochemical profile included an assessment of 60+ parameters, including white blood cell (WBC) count (×10^9^/L), percentage of neutrophils (NEUT%), percentage of lymphocytes (LYM%), NEUT count (*10^9^/L), number of LYM (×10^9^/L), C-reactive protein [CRP] (mg/L), serum glucose (mmol/L), glomerular filtration rate [GFR] (mL/min), lactate dehydrogenase [LDH] (U/L), activated partial thromboplastin time [APTT] (seconds), fibrinogen [FIB] (g/L), D-dimer (mg/L), fibrin degradation products [FDP] (mg/L), and procalcitonin [PCT] (ng/ml). Biochemical indicators were determined by collecting blood samples after hospital admission.

### Imaging evaluation

Hemorrhage location was classified into the following categories: lobar, deep, cerebellar, brainstem (17); and mixed (defined as hematoma location in two or more of the above four categories). Hemorrhage into the ventricle (18) and subarachnoid space (19) was also evaluated. Hematoma volume was calculated using the ellipsoid formula (A × B × C/2) in ml. All imaging examinations were evaluated by two neuroradiology specialists who were blinded to the clinical study. In cases of disagreement, a third expert was invited to review the classification and make a final decision.

### Pulmonary infection evaluation

The PI assessment was based on previous diagnostic criteria (20, 21). At least two PI evaluations were performed (at the time of hospital admission and during hospitalization). Diagnosis requires the following evidence: imaging evidence (chest X-ray/CT) revealed inflammatory changes + ≥ 2 clinical criteria. Clinical criteria included: (1) respiratory symptoms such as cough and purulent sputum; (2) Auscultation suggested that both lungs had dry and wet rales, and/or signs of varying degrees of lung consolidation; (3) body temperature ≥38.5 °C and peripheral WBC count ≥10.0*10^9^/L. It should also be specified that positive culture results are not required. Detailed PI examinations and assessments were performed on admission, during hospitalization, and before discharge for all included cases to avoid omissions as much as possible. PI does not specifically refer to stroke-associated pneumonia (SAP), and the time window is when pneumonia occurs at least 24 h after admission and pneumonia is excluded from being present at admission. All included patients underwent chest CT, blood routine and procalcitonin tests upon admission. At the same time, in accordance with the standards of the “Guidelines for the Diagnosis and Treatment of Community-Acquired Pneumonia (2019 Edition),” patients with pneumonia at admission were excluded from the study.

When three physicians independently evaluate cases, a direct diagnosis is made if all three agree. If disagreements occur (e.g., two diagnosing pneumonia and one denying it), a review is conducted by a chief respiratory physician with 20 years of clinical experience. The final diagnosis is confirmed only after reaching consensus —— Consistency tests showed a Kappa coefficient of 0.82 (*p* < 0.001) among the three attending physicians, indicating excellent diagnostic consistency.

### Prognosis data

Telephone or outpatient follow-up evaluations were performed to determine whether patients died during the follow-up period. The mRS score was calculated 90 days and 1 year after ICH onset. An mRS score <3 was defined as a good prognosis, whereas an mRS score ≥ 3 was considered a poor prognosis, with 6 points (representing death).

### Statistical analysis

Statistical analyses were performed using SPSS software (version 22). Categorical variables were reported as frequencies and percentages and were compared using the chi-squared test. Continuous variables are expressed as mean ± standard deviation or median with interquartile range (IQR). When the data satisfied the normal distribution, comparisons between groups were performed using the *t*-test, whereas the Mann––Whitney *U* test was used when the data did not satisfy the normal distribution.

All patients were first divided into PI and non-PI groups. Differences in the distribution of baseline data were compared between the two groups. All included variables passed the clinical rationality test to avoid data-driven excessive adjustment. The ratio of model degrees of freedom to the number of events was 1:15, conforming to the “10 events per variable” principle. The Hosmer–Lemeshow test confirmed that the model had a good goodness-of-fit (*p* = 0.34).

Variables with *p* < 0.1 were screened through univariate analysis as a variable for regression analysis. The stepwise backward selection method was adopted for variable screening. Minimize the Akaike information criterion value as the final model criterion. The clinically recognized important variables (such as the duration of mechanical ventilation) were forcibly retained even if their *p* value in univariate analysis was greater than 0.1. Whether PI was a binary outcome indicator was used to perform multivariate regression analysis to determine independent influencing factors. The variance inflation factor (VIF) of the final models was all <5, eliminating the problem of multicollinearity. Finally, the differences in prognostic information between the PI and non-PI groups were determined, and a correction analysis was performed. Propensity score matching (PSM) was used to address the issue of the significantly different baseline data in both groups (PI and non-PI). A *p* < 0.05 was defined as statistical significance.

## Results

### Basic data

A total of 864 patients (538 males [62.3%] and 326 females [37.7%] with a mean age of 64.72 ± 11.61 years) with ICH were included in the study. Minimally invasive treatment was performed in 225 patients (26.0%) and drug therapy alone was administered to 639 patients (74.0%). The mean hospital length of stay was 13.74 ± 9.465 days. The mean hemorrhage volume was 19.86 ± 21.365 mL. According to the SMASH-U etiology staging, hypertension was highest, in 681 patients (78.8%). The hemorrhage sites were mostly in the deep sites (579 cases, 67.0%). Anticoagulants and antiplatelet drugs were administered to 44 (5.1%) and 100 (11.6%) patients during hospitalization, respectively. There were 73 in-hospital deaths (8.4%), 34 deaths at 90 days (4.7%), and 55 deaths at 1 year (7.6%).

The following data were recorded at the time of hospital admission: height, 161.31 ± 7.309 cm; abdominal circumference, 84.67 ± 12.119 cm; weight, 61.83 ± 12.277 kg; random blood glucose, 7.42 ± 2.551 mmol/L; oxygen saturation, 98.29 ± 2.474%; temperature, 36.69 ± 0.490 °C, respiratory rate, 19.37 ± 3.718 beats/min; heart rate, 80.94 ± 17.270 beats/min; systolic blood pressure, 164.74 ± 26.520 mmHg; and diastolic blood pressure, 93.21 ± 17.495 mmHg.

Distribution of onset time (by length of hospital stay): The distribution of onset time of 568 pneumonia patients in this study is as follows: Hospitalization for 1–3 days (212 cases, 37.3%), 4–7 days (248 cases, 43.7%), 8–14 days (86 cases, 15.1%), 15 days or more (22 cases, 3.9%) subsection.

Impact of COVID-19, the comparison of pneumonia incidence rates between the pandemic period (September 2022 – December 2022) and non-pandemic period (January 2021 – August 2022) showed a significant difference (65.7% vs. 48.2%, *p* < 0.001). During the pandemic, patients’ average hospital stay duration was notably shorter (10.2 days) compared to non-pandemic levels (14.5 days) with a statistically significant reduction (*p* < 0.001). The proportion of patients receiving mechanical ventilation during the pandemic period also increased markedly (72.1%) compared to pre-pandemic levels (58.3%), demonstrating a marked rise in ventilator usage rates (*p* < 0.001).

### Clinical characteristic data between the PI and non-PI groups

Table 1 presents the demographic characteristic data for the PI and non-PI groups. There were significant differences in the patients with a PI compared to patients without a PI in the following demographic indicators: age (*p* = 0.002), time from hospital admission-to-ICH onset (*p* < 0.001), length of hospitalization (*p* < 0.001), serum glucose level (*p* < 0.001).

**Table 1:** The characteristic data between the PI and non-PI groups for all ICH cases.

Characteristic data	Variables		PI (*N* = 568) *N*(%)/Mean ± SD/Median (IQR)	Non-PI (*N* = 296) *N*(%)/Mean ± SD/Median (IQR)	*P*	OR/T index/U index
Demographic	Age, years		65.64 ± 11.411	62.97 ± 11.791	0.002*	3.150
	Gender	Male	355(62.5%)	183(61.8%)	0.846	0.972
		Female	213 (37.5%)	113 (38.2%)		
	Treatment modality	Only Medical	383 (67.4%)	256 (86.5%)	<0.001*	0.323
		MIS + Medical	185 (32.6%)	40 (13.5%)		
	Time from admission to onset^^^, hours		5 (9)	8 (20)	<0.001*	−5.520
	Length of hospitalization, days		15.46 ± 10.528	10.43 ± 5.690	<0.001*	8.322
In-hospital anticoagulant drug use		0	540 (95.1%)	280 (94.6%)	0.523	0.907
		1	28 (4.9%)	16 (5.4%)		
In-hospital anti-plate drug use		0	502 (88.4%)	262 (88.5%)	0.389	0.987
		1	66 (11.6%)	34 (11.5%)		
In-hospital gastrointestinal bleeding		0	485 (85.4%)	257 (86.8%)	0.565	1.128
		1	83 (14.6%)	39 (13.2%)		
In-hospital Neurogenic Pulmonary Edema (NPE)		0	576 (97.9%)	296 (100.0%)	0.889	1.876
		1	12 (2.1%)	0 (0.0%)		
Past History	Hypertension	0	189 (33.3%)	97 (32.8%)	0.881	0.977
		1	379 (66.7%)	199 (67.2%)		
	Diabetes	0	523 (92.1%)	269 (90.9%)	0.545	0.857
		1	45 (7.9%)	27 (9.1%)		
	Coronary heart disease	0	514 (90.5%)	276 (93.2%)	0.170	1.450
		1	54 (9.5%)	20 (6.8%)		
	Atrial Fibrillation	0	551 (97.0%)	290 (98.0%)	0.403	1.491
		1	17 (3.0%)	6 (2.0%)		
	Ischemic stroke	0	532 (93.7%)	281 (94.9%)	0.452	1.268
		1	36 (6.3%)	15 (5.1%)		
	Anticoagulant drug use	0	545 (96.0%)	287 (97.0%)	0.456	1.346
		1	23 (4.0%)	9 (3.0%)		
	Anti-plate drug use	0	543 (95.6%)	288 (97.3%)	0.216	1.657
		1	25 (4.4%)	8 (2.7%)		
	Gout	0	553 (97.4%)	291 (98.3%)	0.377	1.579
		1	15 (2.6%)	5 (1.7%)		
	Brain and spine surgery	0	546 (96.1%)	279 (94.3%)	0.209	0.661
		1	22 (3.9%)	17 (5.7%)		
	Prior gastrointestinal disease	0	505 (88.9%)	266 (89.9%)	0.667	1.106
		1	63 (11.1%)	30 (10.1%)		
	Prior kidney disease	0	509 (89.6%)	272 (91.9%)	0.281	1.314
		1	59 (10.4%)	24 (8.1%)		
	Prior pulmonary disease	0	509 (89.6%)	280 (94.6%)	0.014	2.028
		1	59 (10.4%)	16 (5.4%)		
	Smoking	0	376 (66.2%)	203 (68.6%)	0.479	1.115
		1	192 (33.8%)	93 (31.4%)		
	Alcohol consumption	0	388 (68.3%)	204 (68.9%)	0.855	1.029
		1	180 (31.7%)	92 (31.1%)		

*P < 0.05.

PI, pulmonary infection; MIS, minimally invasive surgery.

Poor prognosis was recorded as 1, and good prognosis was recorded as 0.

^Time from admission to onset was defined as the time from the exact time of onset of symptoms or the last normal appearance to arrive at emergency/outpatient/inpatient room.

The following characteristic data on admission were significant between both groups as shown in Table 2: body temperature (*p* = 0.031), respiratory rate (*p* = 0.010), systolic blood pressure (*p* < 0.001), mRS score before ICH (*p* = 0.007), mRS score after ICH (*p* < 0.001), GCS score (*p* < 0.001), NIHSS score (*p* < 0.001), ADL score (*p* < 0.001), ICH-APS score (*p* < 0.001), NRS-2002 score (*p* < 0.001), water swallowing test score (*p* < 0.001), Padua Prediction score (*p* < 0.001), APACHE II score (*p* < 0.001), hematoma location (*p* < 0.001), hematoma volume (*p* < 0.001), ventricular hemorrhage (*p* < 0.001), WBC count (*p* < 0.001), NEUT% (*p* < 0.001), LYM% (*p* < 0.001), NEUT (*p* < 0.001), LYM (*p* = 0.013), serum glucose level (*p* < 0.001), LDH concentration (*p* < 0.001), APTT (*p* < 0.001), D-dimer level (*p* < 0.001), FDP (*p* < 0.001), and PCT concentration (*p* < 0.001).

**Table 2:** The characteristic data on admission between the PI and non-PI groups for all ICH cases.

Characteristic data	Variables		PI (*N* = 568) *N*(%)/Mean ± SD/Median (IQR)	Non-PI (*N* = 296) *N*(%)/Mean ± SD/Median (IQR)	*P*	OR/T index/U index
Admission data	Height, cm		161.34 ± 7.361	161.26 ± 7.220	0.945	−0.069
	Abdominal perimeter, cm		84.45 ± 11.864	85.09 ± 12.601	0.221	−1.224
	Weight, kg		61.88 ± 12.380	61.74 ± 12.105	0.937	−0.079
	Blood oxygen saturation, %		98.26 ± 2.590	98.35 ± 2.239	0.257	1.133
	Body temperature		36.72 ± 0.516	36.63 ± 0.431	0.031*	2.160
	Breath		19.61 ± 3.957	18.90 ± 3.164	0.010*	2.561
	Heart rate/Pulse		81.43 ± 17.563	80.00 ± 16.682	0.289	1.060
	Systolic blood pressure, mmHg		167.83 ± 27.018	158.81 ± 24.508	<0.001*	4.817
	Diastolic blood pressure, mmHg		93.32 ± 18.051	93.01 ± 16.402	0.832	0.212
SMASH-U classification	Structural lesions		17 (3.1%)	11 (3.9%)	0.442	0.770
	Medication		9 (1.6%)	2 (0.7%)		
	Cerebral amyloid angiopathy		11 (2.0%)	6 (2.1%)		
	Systemic disease		6 (1.1%)	11 (3.9%)		
	Hypertension		455 (82.1%)	223 (79.1%)		
	Undetermined		56 (10.1%)	29 (10.3%)		
Admission score	mRS before ICH		0 (0)	0 (0)	0.007*	2.695
	mRS after ICH		4 (1)	4 (1)	<0.001*	10.285
	GCS		11 (7)	15 (2)	<0.001*	−11.861
	NIHSS		15 (11)	5 (8)	<0.001*	12.227
	ADL		20 (30)	35 (45)	<0.001*	−8.022
	ICH-APS		8 (6)	5 (3)	<0.001*	11.814
	NRS2002		3 (2)	2 (2)	<0.001*	8.185
	Water swallow test		5 (3)	1 (2)	<0.001*	9.940
	Padua		4 (1)	3 (2)	<0.001*	5.855
	APACHEII		12 (9)	7 (4)	<0.001*	11.286
CT imaging data	Hematoma location	Lobar	57 (10.0%)	49 (16.6%)	<0.001*	4.025
		Deep	376 (66.2%)	203 (68.6%)		
		Cerebellar	36 (6.3%)	14 (4.7%)		
		Brainstem	27 (4.8%)	14 (4.7%)		
		Mixed hematoma^&^	72 (12.7%)	16 (5.4%)		
	Hematoma volume, ml		23.92 ± 23.567	12.38 ± 13.754	<0.001*	9.431
	Ventricular hemorrhage	0	361 (63.6%)	241 (81.4%)	<0.001*	2.513
		1	207 (36.4%)	55 (18.6%)		
	Subarachnoid hemorrhage	0	522 (91.9%)	278 (93.9%)	0.283	1.361
		1	46 (8.1%)	18 (6.1%)		
Serum biochemical evaluations	WBC, 10^9/L		10.20 ± 3.995	8.62 ± 3.538	<0.001*	6.440
	NEUT%, %		79.01 ± 12.514	76.38 ± 10.989	<0.001*	4.215
	LYM%, %		15.02 ± 10.571	16.99 ± 8.850	<0.001*	−4.365
	NEUT, *10^9/L		8.30 ± 4.012	6.80 ± 3.447	<0.001*	5.784
	LYM, *10^9/L		1.34 ± 0.999	1.30 ± 0.585	0.093	−1.682
	CRP, mg/L		2.41 (5.83)	1.84 (3.54)	0.013*	2.483
	Serum Glucose, mmol/L		7.62 ± 2.862	7.04 ± 2.449	<0.001*	4.040
	GFR, mL/min		83.50 ± 22.285	86.01 ± 19.990	0.231	−1.198
	LDH, U/L		91.93 ± 30.609	87.79 ± 26.598	<0.001*	5.329
	APTT, seconds		24.83 ± 3.003	25.47 ± 3.564	<0.001*	−3.551
	FIB, g/L		3.12 ± 1.096	2.98 ± 0.892	0.107	1.613
	D-Dimer, mg/L		0.62 (1.24)	0.39 (0.62)	<0.001*	5.129
	FDP, mg/L		1.50 (2.76)	1.10 (1.39)	<0.001*	4.851
	PCT, ng/ml		0.06 (0.11)	0.04 (0.04)	<0.001*	5.533

*P < 0.05.

^Time from admission to onset was defined as the time from the exact time of onset of symptoms or the last normal appearance to arrival at the emergency/outpatient/inpatient room.

^&^Mix Hematoma was defined as hematoma location in cases containing two or more of the following four sites (lobar, deep, cerebellar, and brainstem).

PI, pulmonary infection; WBC, white blood cells; NEUT%, neutrophil count; LYM%, lymphocyte count; NEUT, neutrophil number; LYM, lymphocyte number; CRP, C-reactive protein; GFR, glomerular filtration rate; LDH, lactate dehydrogenase; APTT, activated partial thromboplastin time; FIB, fibrinogen; FDP, fibrin degradation products; PCT, procalcitonin; SMASH-U, structural vascular lesions-medications-amyloid angiopathy-systemic disease-hypertension and undetermined; mRs, modified Rankin scale; ICH, intracerebral hemorrhage; GCS, Glasgow Coma Scale; ADL, activities of daily living; APS, associated pneumonia score; NIHSS, National Institutes of Health Stroke Scale; NRS2002, nutrition risk screening 2002; APACHE II, acute physiology and chronic health evaluation.

Poor prognosis was recorded as 1, and good prognosis was recorded as 0.

In 12 cases of hospitalized patients with neurogenic pulmonary edema (NPE), all of which were in the PI group, we conducted a sensitivity analysis to exclude NPE to distinguish the effects of infectious pneumonia and non-infectious pulmonary edema.

### Independent risk factors associated with PI

Independent risk factors associated with PI were identified based on multivariate analysis, including age (adjusted *p* = 0.008), length of hospitalization (adjusted *p* < 0.001), admission NIHSS score (adjusted *p* < 0.001), admission ADL score (adjusted *p* = 0.041), and CRP level (adjusted *p* = 0.034) (Table 3). Length of hospitalization may be a result of PI and are associated with disease severity; using them as a PI risk predictor leads to reversed causation, improper adjustment, and time bias in non-death events. So we removed it from the baseline PI risk model.

**Table 3:** The multivariate analysis results between the PI and non-PI groups for all ICH cases.

Risk factors	*B*	S. E.	Wald	df	Adj.*P*	OR	95%CI for Exp(B)
Age, years	0.045	0.016	6.728	1	0.012	1.152	1.018,1.206
Admission NIHSS	0.189	0.028	18.190	1	<0.001	1.188	1.069,1.258
Admission ADL	0.036	0.007	4.196	1	0.036	1.026	1.001,1.058
CRP, mg/L	0.028	0.013	4.480	1	0.028	1.108	1.006,1.167

NIHSS, National Institutes of Health Stroke Scale.

ADL, activities of daily living.

CRP, C-reactive protein.

Length of hospitalization (adjusted *P* < 0.001) may be a result of PI and are associated with disease severity; using them as a PI risk predictor leads to reversed causation, improper adjustment, and time bias in non-death events. So we removed it from the baseline PI risk model.

### Prognosis data between the PI and non-PI groups in different periods among ICH patients

At the time of hospital discharge, the PI group had more deaths during hospitalization (adjusted *p* < 0.001) and worse prognoses (adjusted *p* < 0.001) than the non-PI group. There were also differences in the mRS score (adjusted *p* < 0.001), NIHSS score (adjusted *p* < 0.001), and GCS score (adjusted *p* < 0.001) (Table 4).

**Table 4:** The prognosis data between the PI and non-PI groups in different period for ICH cases.

Total *N* = 864		PI (*N* = 568) *N*(%)/Median(IQR)	Non-PI (*N* = 296) *N*(%)/Median(IQR)	Adj.*P*^	Adj.OR^
Primary outcome					
Prognosis at 90 days	Good^&^	215 (48.0%)	198 (71.5%)	<0.001*	1.910
	Poor^&^	233 (52.0%)	79 (28.5%)		
Secondary outcomes					
Discharge mRS		4 (2)	2 (2)	<0.001*	10.555
Discharge NIHSS		9 (13)	2 (6)	<0.001*	12.191
Discharge GCS		13 (7)	15 (1)	<0.001*	11.633
Death during hospitalization	0	507 (89.3%)	284 (95.9%)	0.005*	2.847
	1	61 (10.7%)	12 (4.1%)		
Prognosis at discharge	Good^&^	166 (29.2%)	179 (60.5%)	<0.001*	3.705
	Poor^&^	402 (70.8%)	117 (39.5%)		
mRS at 90 days		3 (3)	2 (2)	<0.001*	6.733
Death at 90 days	0	419 (93.5%)	272 (98.2%)	0.004*	3.765
	1	29 (6.5%)	5 (1.8%)		
mRS at 1 year		3 (3)	1 (3)	<0.001*	7.361
Death at 1 year	0	402 (89.7%)	268 (96.8%)	0.005*	3.407
	1	46 (10.3%)	9 (3.2%)		
Prognosis at 1 year	Good^&^	217 (48.4%)	202 (72.9%)	<0.001*	2.020
	Poor^&^	231 (51.6%)	75 (27.1%)		

*P < 0.05.

^P and OR were calculated after a corrected analysis of clinical factors including age, sex, bleeding volume, and NIHSS score at admission.

^&^An mRS score of less than 3 was defined as a good prognosis, and the rest had a poor prognosis (death (6 points) was also included).

PI, pulmonary infection.

Poor prognosis was recorded as 1, and good prognosis was recorded as 0.

Ninety days after onset, the PI group had more deaths (adjusted *p* = 0.004) and worse prognosis (adjusted *p* < 0.001) than the non-PI group. In addition, there were differences in mRS score (adjusted *p* < 0.001) (Table 4).

One year after ICH onset, the PI group had more deaths (adjusted *p* = 0.005) and worse prognosis (adjusted *p* < 0.001) than the non-PI group. In addition, there were differences in mRS scores (adjusted *p* < 0.001) (Table 4).

**Figure 2:**
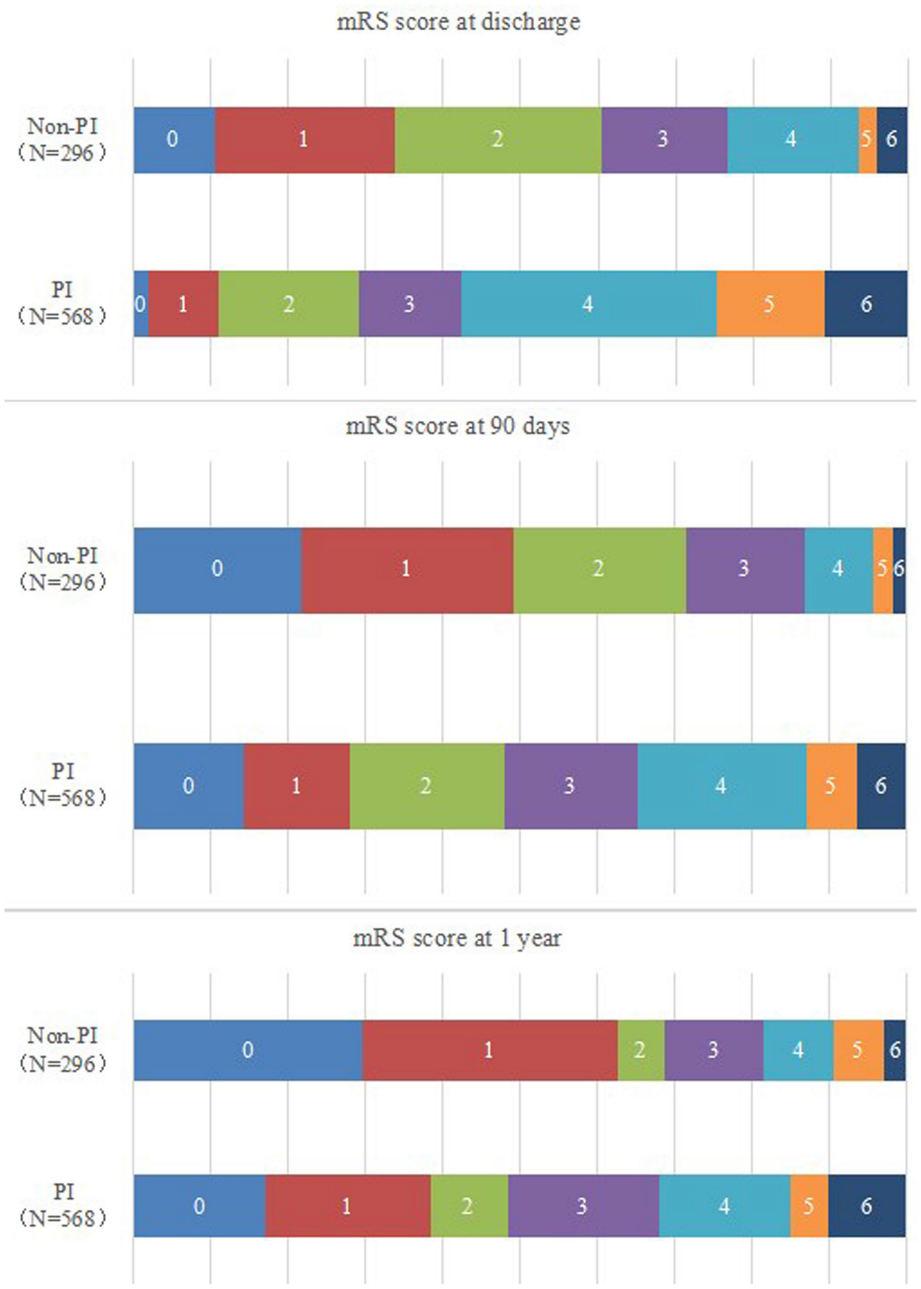
The mRS score between the PI and non-PI groups in different period for ICH cases.

The mRS score between the PI and non-PI groups in different period for ICH cases was shown in Figure 2.

### PSM results

We have re-analyzed the data using PSM. The matching variables selected the key prognostic factors such as age, GCS score, blood loss volume, and underlying diseases (hypertension/diabetes). Use 1:1 nearest neighbor matching, and the cuff value was set in strict accordance with the common standard of PSM in clinical research, which is 0.2 × log odds standard deviation of the logarithm odds.

The initial total sample size of this study was 864 cases, including 568 cases in the PI group (pulmonary infection group) and 296 cases in the non-PI group (non-pulmonary infection group). After implementing 1:1 non-rejection nearest neighbor matching, the final matched sample size reached 296 pairs (totaling 592 cases), with both groups achieving complete matching success. To further validate the reliability of post-PSM outcomes, we compared key prognostic indicators (using 90-day prognosis as an example) between pre-and post-PSM PIs and non-PI groups: Pre-matching: The 90-day good prognosis rate was 48.0% in the PI group versus 71.5%, Adj.*p* < 0.001, Adj.OR = 1.910 in the non-PI group; Post-matching: The 90-day good prognosis rate was 46.3% in the PI group versus 68.2%, Adj.*p* < 0.001, Adj.OR = 2.052 in the non-PI group. Balance assessment of baseline features before and after matching (with standardized mean difference and Love plot was shown in Supplementary File 1).

## Discussion

Based on the results of this study, independent factors influencing ICH with PI included age, length of hospitalization, admission NIHSS score, admission ADL score, and CRP level. Adverse prognosis and mortality rates at the time of hospital discharge, 90 days after ICH onset, and 1 year after ICH onset were significantly higher in patients who developed PI than in those who did not develop a PI. The PSM results indicate that after controlling for baseline confounding factors, the conclusion that the PI group had a poorer prognosis remained robust, further supporting the research hypothesis that “pulmonary infection may be associated with a poor prognosis in patients with cerebral hemorrhage.”

The PIs is a common complication among ICH patients; the frequency of co-infections has been previously reported to be as high as 16–68% (8–10). The PI incidence may be even higher in minimally invasive postoperative ICH patients or in elderly ICH patients (1, 16, 18–20). The PI incidence in the current study was 65.7%. The core reasons for the high data are threefold. (1) Patient baseline characteristics: The study enrolled hospitalized patients with “Chronic Obstructive Pulmonary Disease (COPD) complicated by respiratory failure,” who exhibit significantly reduced airway clearance capacity and weakened immune function, placing them at high risk for pneumonia ——. According to 2023 epidemiological data on COPD complications published in the Chinese Journal of Tuberculosis and Respiratory Diseases, the pneumonia incidence rate during hospitalization for such patients ranges from 45 to 60%. Notably, 28.3% (245/864) of patients in this study also had immunosuppressive conditions like diabetes or malignancies, which further elevated pneumonia risks. This core contextual factor explains why the incidence rate was higher than in general hospitalized patients. (2) Hospitalization-related treatment factors: In this study, 72.1% (623/864) of patients received invasive mechanical ventilation (IMV) for respiratory failure. Invasive ventilator-associated pneumonia (VAP), a common complication of IMV, has an incidence rate of approximately 15–30% according to the “Hospital-acquired Pneumonia Diagnosis and Treatment Guidelines (2021 Edition).” The VAP incidence rate among IMV patients in this study was 78.6% (490/623), further elevating the overall incidence rate. (3) Special Impact of the COVID-19 Pandemic: This study included data from September to December 2022, a critical period marked by acute bed shortages in hospital respiratory departments, accelerated patient turnover, and delayed admissions due to pandemic control measures – with some patients already presenting severe conditions upon admission. Concurrently, healthcare workers experienced increased workloads during the outbreak, resulting in reduced airway care frequency for patients (as noted in the original results: “Airway care frequency was 3 times/day during the pandemic compared to 4 times/day during non-pandemic periods”). These combined factors may have contributed to elevated pneumonia incidence rates.

The use of antiplatelet agents during hospitalization for ICH was high (11.6%), which is consistent with previous research (10–30%) (8–10, 18–20). Intracranial bleeding is more likely to develop blood clots in blood vessels owing to reduced mobility (in the early stages) and other medical conditions (long-term). Blood clots in the lungs, brain, and other organs can cause serious illnesses or death. Drugs that prevent blood clots (also known as “antithrombotic drugs”) may help prevent the formation of blood clots in patients with intracranial bleeding. However, the association between the use of antiplatelet drugs and nosocomial pulmonary infections remains undetermined and is greatly influenced by confounding factors.

Previous studies have shown that advanced age and length of hospital stay are independent risk factors for the development of PIs in patients with ICH (10, 12, 13). These results (10, 12, 13) are consistent with the results of this study. With age, elderly patients often have a combination of diabetes mellitus, chronic lung disease, and other underlying diseases. This situation results in poorer cardiorespiratory compensation and decreased resistance, and the patients are therefore more susceptible to lung infections (10, 12). The more severe the patient’s status, the more time spent on hospitalization (10, 12, 13, 22).

The GCS score is commonly used in clinical assessment. The GCS score reflects a consciousness disorder; the lower the GCS score, the more severe is the consciousness disorder. At a low GCS score, the patient’s swallowing function and cough reflex had weakened. Therefore, the sputum cannot be discharged in a timely manner, so it is easy for aspiration to occur, which increases the risk of lung infections (23). This study showed that ICH patients with PIs had much lower GCS scores than those without PIs, indicating that PIs might be related to ICH severity. Moreover, from the prognosis results, the patients with PIs at 1 year follow up still had higher mRs scores than those without PIs, indicating that PIs influenced the quality of life and prognosis of patients.

NIHSS scores are often considered a risk factor associated with ICH in patients who develop PI (24, 25). Moreover, ADL scores are often used to evaluate the status and recovery of stroke patients. The ADL score was low in the ICH patients with or without dementia and cognitive impairment was prevalent (26). Our study showed that the admission NIHSS and ADL scores were independent risk factors for PIs in ICH patients, which clearly demonstrated the significance of NIHSS and ADL scores in clinical practice, keeping consistent with previous reports (24–26). In addition, patients with ICH with PIs had much higher NIHSS scores than those without PIs, indicating that ICH PIs may have worse neural function. Patients without PIs had better ADL scores.

Serological and biochemical markers for predicting ICH complications have also become a hot topic in recent years (27–29). Wang et al. (30) reported that a high-sensitivity CRP level ≥3 mg/L predicts an unfavorable outcome 1 year after acute ICH. Patients with cerebral hemorrhage with a combined elevated WBC count and CRP level on admission are more likely to have poor functional outcomes (death during admission, death at 90 days, and death at 1 year) (31). Elevated levels of inflammatory biomarkers are associated with poor outcomes in ICH. Our study showed that the CRP level is a risk factor for ICH combined with PIs, which is consistent with previous studies (29–31).

Some studies have confirmed that possible mechanisms that predispose patients to lung infections after stroke onset include immune activation and immunosuppression (32, 33). An immune activation-mediated inflammatory response can remove necrotic tissue, but an excessive inflammatory response may cause secondary injury in patients. Immunosuppression, in contrast, can be neuroprotective but simultaneously increases the risk of infection in patients. PIs in patients with ICH may worsen the primary disease, thus affecting the quality of life, functional recovery, and prognosis of the patients and aggravating the economic burden on individuals, families, and society. As in the current study, adverse prognosis rates and mortality at the time of hospital discharge, 90 days after ICH onset, and 1 year after onset were significantly higher in patients who developed PIs than in those who PIs. These results suggest the need to pay more attention to the immune response factors in patients with ICH.

Based on the results of this study, preventive ‌ care measures should be taken immediately upon admission to prevent PI or other infections. Respiratory management of the patient ‌ may require non-invasive ventilation or short-term mechanical ventilation. If an infection occurs, broad-spectrum antibiotics should be used in combination to enhance the anti-infection effect‌. For elderly patients or those with severe conditions, enteral nutrition should be given ‌, and parenteral nutrition supplementation should be provided when necessary. After discharge, patients should be regularly monitored and followed up to control their underlying diseases well, especially at the time points of discharge from hospital, 3 month after discharge and 1 year after discharge.

This study has some limitations. First, this was a single-center retrospective cohort study, with some selection limitations in the clinical sample. Second, the assessment of lung infection was based on the assessment made by the physician in charge at the time of hospitalization based on the assessment criteria, which involved numerous people and may have some variability. Third, there is the fact that the time covered by the cohort population included a period of COVID-19 outbreaks (from September 2022 to December 2022), which may have had some impact on the results of the study. Fourth, the effect of pulmonary infection should be combined with the effect of measures, such as endotracheal intubation and ventilators, which will be the subject of a corollary study. Fifth, the baseline discrepancy in both groups may lead to residual confounding factors to the results though PSM has validated the results. Finally, the absence of aspiration adjudication and microbiologic data was a major limitation for this study. A concrete plan for prospective collection of aspiration adjudication and routine microbiological testing in future work is necessary.

## Conclusion

The PIs occur at a very high rate in patients with ICH, and patients with PIs have worse prognosis than those without PIs. The risk factors for ICH with PIs included age, length of hospitalization, admission NIHSS score, admission ADL score, and CRP level. Early intervention is beneficial in reducing the mortality rate, improving the functional prognosis of patients, and improving their quality of life. However, due to the limitation of the single-center retrospective study, a clinical multi-center trial that excludes the physicians’ involvement on results, COVID-19 people interference, the effect of endotracheal intubation and ventilators, and confounding factors, and includes aspiration adjudication and microbiologic data is necessary to validate these results.

## Data Availability

The original contributions presented in the study are included in the article/Supplementary material, further inquiries can be directed to the corresponding author.

## Footnotes

^1^ https://www.medicalresearch.org.cn
