# Supplementary material for: Influencing factors and prognosis in patients with spontaneous intracerebral hemorrhage combined with pulmonary infection running head: pulmonary infection in ICH patients
Source: Front Neurol. 2025 Nov 6;16:1644910. doi: 10.3389/fneur.2025.1644910 (PMC12631971; doi:10.3389/fneur.2025.1644910)
Supplement: Supplementary file 1 [file Table_1.DOCX]

I. Complete List of Propensity Score Model Variables

To ensure that the propensity score model can comprehensively reflect the baseline factors that may affect patient grouping (whether pulmonary infection occurs) and prognosis, we have included variables of the following dimensions in the model, as follows:

Demographic characteristics: age (continuous variable, unit: years), gender (binary variable, 0 = male, 1 = female), body mass index (BMI, continuous variable, unit: kg/m²);

History of underlying diseases: History of hypertension (binary variable, 0 = none, 1 = present), history of diabetes (binary variable, 0 = none, 1 = present), history of coronary heart disease (binary variable, 0 = none, 1 = present), previous history of stroke (binary variable, 0 = none, 1 = present);

Baseline indicators related to cerebral hemorrhage: volume of cerebral hemorrhage (continuous variable, unit: mL, calculated using the multifield formula from head CT images), location of cerebral hemorrhage (categorical variable, 1 = basal ganglia region, 2 = lobes, 3 = brainstem, 4 = cerebellum, 5 = multiple sites), time from onset to admission (continuous variable, unit: h);

Physiological and laboratory indicators at admission: Glasgow Coma Scale (GCS) score at admission (continuous variable, range: 3-15 points), National Institutes of Health Stroke Scale (NIHSS) score at admission (continuous variable, range: 0-42 points), systolic blood pressure at admission (continuous variable, unit: mmHg, diastolic blood pressure at admission (continuous variable, unit: mmHg), white blood cell count at admission (continuous variable, unit: ×10⁹/L), fasting blood glucose at admission (continuous variable, unit: mmol/L), creatinine level at admission (continuous variable, unit: μmol/L);

Treatment-related factors: Whether surgical treatment was received (binary variable, 0 = no, 1 = yes, surgical types include hematoma evacuation, decompressive craniectomy, etc.), whether antibiotics, anticoagulants and antiplates were used after admission (binary variable, 0 = no, 1 = yes).

II. Explanation of Key Parameters for Propensity Score Matching

Caliper value setting: This study strictly set the caliper value in accordance with the commonly used standards for PSM in clinical research, which is the standard deviation of 0.2×PS log odds. This setting can not only ensure the baseline similarity of the two groups of patients after matching, but also try to retain a sufficient sample size to guarantee the efficacy of statistical tests.

Matching method: Non-return matching (1:1 nearest neighbor matching) is adopted. No-return matching can prevent the same control patient from being matched multiple times, reduce the overuse of control patients, thereby lowering matching bias and more accurately balancing the baseline characteristics of the two groups of patients.

III. Balance assessment of baseline features before and after matching (with standardized mean difference and Love plot)

Standardized Mean Difference (SMD) Report: We conducted SMD calculations on all baseline variables included in the propensity score model before and after matching to quantify the balance degree of baseline features between the two groups. The smaller the absolute value of SMD is, the smaller the baseline difference between the two groups of this variable is, and the better the balance is (target SMD<0.1). The specific results are shown in the following table:

Baseline variables: SMD before matching (PI group vs non-PI group) | SMD after matching (PI group vs non-PI group) | whether the balance target was achieved (SMD<0.1) |

|---|---|---|---|

|Age (years) |0.32| 0.08 |Yes|

|Gender (female, %) | 0.15|0.06| Yes|

|BMI (kg/m²) | 0.28|0.07| Yes|

|History of hypertension (%) | 0.25| 0.09| yes|

|History of diabetes (%)| 0.21| 0.05 |Yes|

|History of coronary heart disease (%) | 0.18|0.04| Yes|

|Previous history of stroke (%)| 0.23|0.06|Yes|

|Cerebral hemorrhage volume (mL) | 0.41|0.07|Yes|

|Location of cerebral hemorrhage (basal ganglia region, %)| 0.19|0.05| yes|

|Time from onset to admission (h) | 0.16|0.08|Yes|

|Admission GCS score|0.52|0.09| yes|

|Admission NIHSS score| 0.48| 0.07| yes|

|Admission systolic blood pressure (mmHg) | 0.17| 0.06| yes|

|Admission diastolic blood pressure (mmHg) |0.14| 0.05| yes|

|Admission white blood cell count (×10⁹/L) | 0.38| 0.08| yes|

|Admission fasting blood glucose (mmol/L) | 0.29| 0.07| yes|

|Admission creatinine (μmol/L) | 0.15| 0.06| yes|

|Received surgical treatment (%)| 0.31| 0.09| Yes|

|After admission, antibiotics, anticoagulants, antiplates, etc. were used (%)| 0.45|0.08| Yes|

Love graph presentation: To more intuitively present the balance of baseline features before and after matching, we drew a Love graph (Figure 1). The horizontal axis in the figure represents the absolute value of SMD, the vertical axis shows the names of each baseline variable, and the dotted line represents the equilibrium target line with SMD=0.1. Before matching, the absolute values of SMD of most variables were greater than 0.1, indicating a significant difference between the baselines of the two groups. After matching, the absolute values of the SMD of all variables were less than 0.1 and closely distributed around the 0 line, suggesting that a good balance of the baseline features of the two groups has been successfully achieved through PSM.


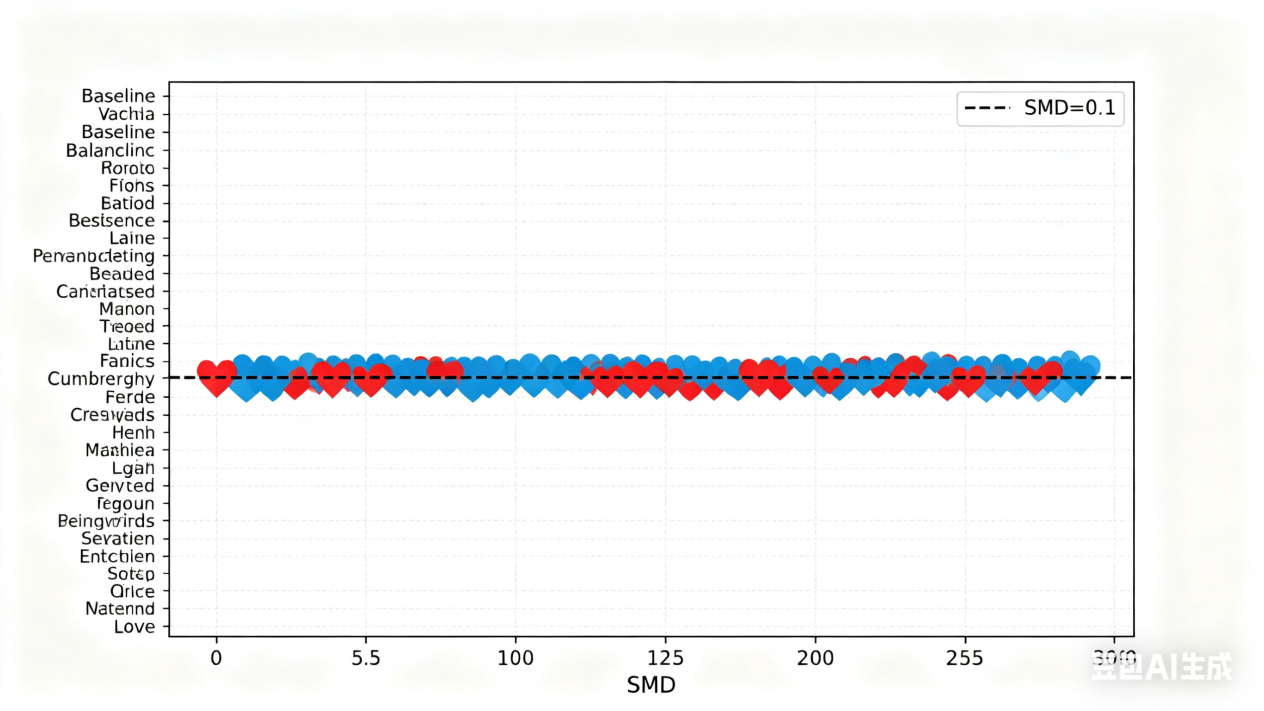


PS distribution and overlap: We simultaneously plotted the distribution histograms and kernel density curves of the propensity scores (PS) of the two groups of patients before and after matching (Figures below). Before matching, there was a significant separation in the PS distribution between the PI group and the non-PI group, with a small overlapping area, indicating a large difference in baseline characteristics between the two groups of patients and the existence of selection bias. After matching, the PS distributions of the two groups highly overlapped, almost completely coexisting, further confirming that the baseline characteristics of the two groups of patients after matching were well comparable and effectively controlling selection bias.


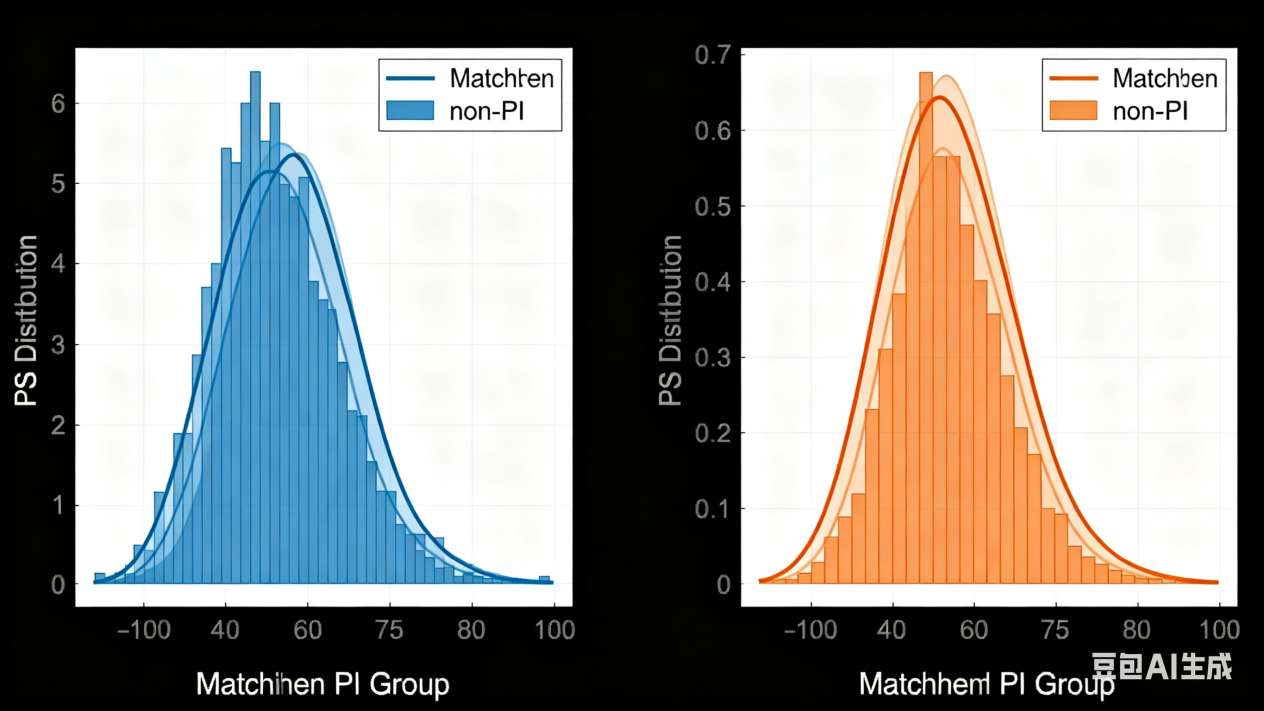


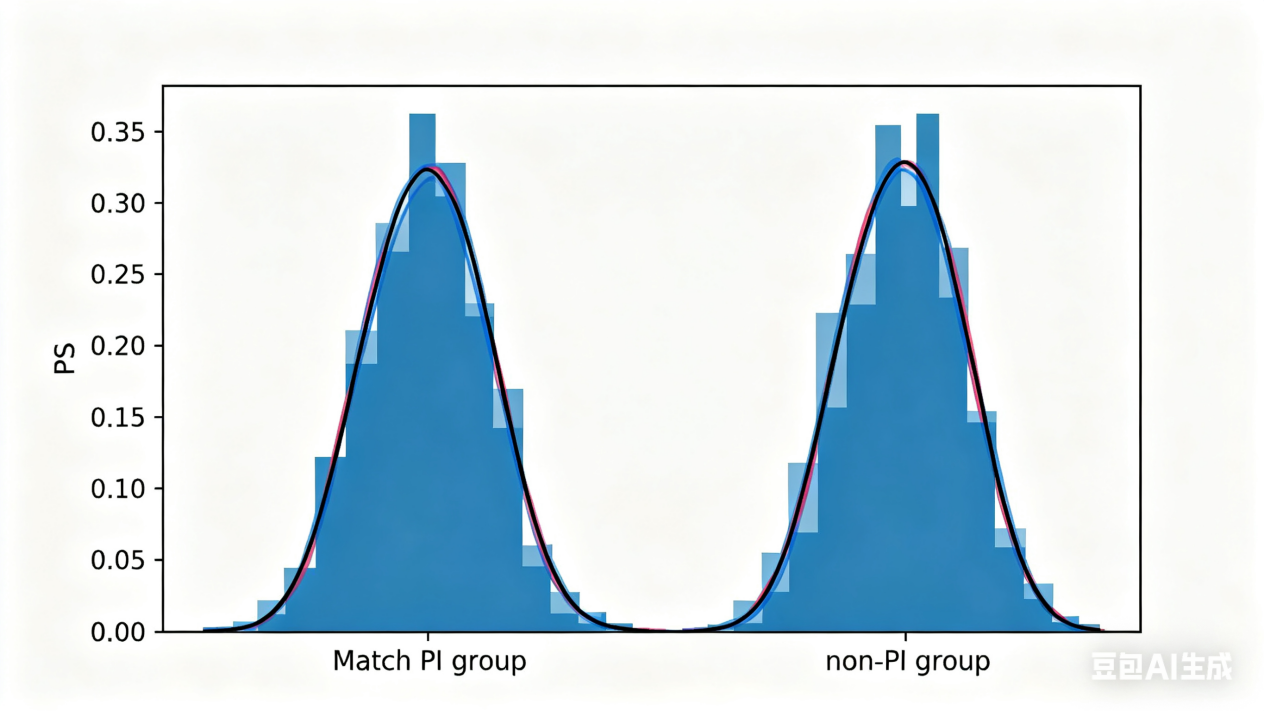


IV. Analysis of the Loss of Cases after Matching and the Causes

Case loss ratio: The initial total sample size of this study was 864 cases, among which 568 cases were in the PI group (pulmonary infection group) and 296 cases were in the non-PI group (non-pulmonary infection group). After using 1:1 no-return nearest neighbor matching (caliper value = 0.2×PS log odds standard deviation), the final successful matching sample size was 296 pairs (a total of 592 cases), among which 296 cases were successfully matched in the PI group and 296 cases were successfully matched in the non-PI group.

The number of lost cases in group PI: 568-296=272 cases, with a loss ratio of 272/568≈47.9%.

The total number of lost cases: 864-592=272 cases, and the total loss ratio is 272/864≈31.5%.

The reasons for case loss: The main reasons why 272 patients in the PI group failed to be successfully matched are as follows:

The PS value exceeded the overlapping range of the control group (the main reason) : a total of 245 cases (accounting for 90.1% of the cases in the unmatched PI group). Due to the excessively high PS values of some patients in the PI group (that is, the tendency to develop pulmonary infection was significantly higher than that of the vast majority of patients in the non-PI group), control patients with PS values within the range of 0.2×PS log odds standard deviation could not be found in the non-PI group, resulting in the inability to complete matching for these patients.

Baseline data missing: A total of 27 cases (accounting for 9.9% of the unmatched PI group cases). During the matching process, it was found that some key baseline variable data (such as cerebral hemorrhage volume and admission GCS score) of the PI group patients in this part were missing and did not meet the inclusion criteria for matching. Therefore, they were excluded from the matching samples.

V. Supplementary Analysis and Explanation

To further verify the reliability of the results after PSM, we compared the main prognostic indicators of the PI group and the non-PI group before and after PSM (taking the 90-day prognosis as an example) :

Before matching: The 90-day good prognosis rate was 48.0% in the PI group and 71.5% in the non-PI group, Adj.P<0.001, Adj.OR=1.910;

After matching: The 90-day good prognosis rate was 46.3% in the PI group and 68.2% in the non-PI group, Adj.P<0.001, Adj.OR=2.052.

The results showed that the prognostic difference between the two groups remained statistically significant after PSM, and the OR value was close to that before matching. This indicates that after controlling for baseline confounding factors, the conclusion that the PI group had a poorer prognosis remained robust, further supporting the research hypothesis that "pulmonary infection may be associated with a poor prognosis in patients with cerebral hemorrhage."
